# Ocular adverse effects of anti-cancer chemotherapy

**DOI:** 10.25122/jml-2023-0041

**Published:** 2023-06

**Authors:** Elena Andreea Stoicescu, Raluca Claudia Iancu, Alina Popa Cherecheanu, George Iancu

**Affiliations:** 1 Faculty of Medicine, Carol Davila University of Medicine and Pharmacy, Bucharest, Romania; 2 Department of Ophthalmology, Emergency University Hospital, Bucharest, Romania; 3 Filantropia Clinical Hospital of Obstetrics and Gynecology, Bucharest, Romania

**Keywords:** ocular adverse effects, chemotherapy, breast cancer, ocular surface

## Abstract

Cancer ranks as the second leading cause of mortality in Europe, following cardiovascular diseases. Every year, 2.6 million people are diagnosed with this disease, and 1.2 million die. It has an impact not only on individual health but also on society and the economy. The survival rate has improved with the introduction of new diagnostic methods and anti-cancer chemotherapy. While more aggressive chemotherapeutic regimens and combination therapies have demonstrated efficacy against cancer cells, they also have detrimental effects on normal cells, leading to systemic and ocular adverse reactions associated with cytotoxicity, inflammation, and neurotoxicity. Consequently, we have an increased survival rate, but the appearance of these ocular adverse effects decreases the quality of life. Ocular toxicity induced by chemotherapeutic agents is often underestimated. While prevention may not be possible, proper management by an ophthalmologist, an integral part of the oncology patient's medical team, is crucial. The ophthalmologist should assess the patient before initiating chemotherapeutic treatment and continue monitoring throughout to identify any adverse ocular reactions resulting from the systemic chemotherapy. This article aimed to briefly highlight the adverse reactions occurring at the ocular surface in patients undergoing chemotherapeutic treatment. Fortunately, these ocular side effects are limited only to the period in which the chemotherapeutic treatment is done, with most of them disappearing a few weeks after stopping the treatment.

## INTRODUCTION

Systemic chemotherapeutic treatment can cause damage to various organs, including the heart, lungs, central nervous system, bones, and bone marrow [1]. With the emergence of more aggressive and combination chemotherapeutic therapies for different stages of cancer, there has been increased recognition of ocular adverse reactions [1]. Many chemotherapeutic drugs have ocular toxicity, resulting in various secondary ocular reactions, which are attributed to the unique physiological and anatomical characteristics of the eye. The ocular surface must maintain constant homeostasis for proper functioning [1]. For combined chemotherapeutic therapies, it is difficult to determine which agent produced a certain ocular adverse effect; therefore, these ocular adverse effects are under-reported. This article provides an overview of chemotherapeutic agents commonly used in oncological patients, which can produce adverse reactions on the ocular surface.

### 1. Alkylating agents

These drugs act by substituting hydrogen atoms in some organic compounds with alkyl groups [1]. Oxaliplatin is used to treat locally advanced pancreatic cancer or metastases, advanced esophageal cancer, advanced ovarian cancer, relapsed non-Hodgkin's lymphoma, palliative treatment of testicular cancer, and advanced colon and rectal cancer. Studies have reported hyper lacrimation, conjunctivitis, decreased visual acuity, and narrowing of the visual field as ocular side effects of this medication [2, 3].

Cyclophosphamide is used as monotherapy or in combination with other antineoplastic drugs for the treatment of leukemias, malignant lymphomas, solid malignant tumors with or without metastases (such as breast cancer, testicular cancer, small cell lung carcinoma, neuroblastoma, Ewing's sarcoma). Ocular manifestations such as keratoconjunctivitis sicca, blepharoconjunctivitis, and reversible epiphora have been observed in patients treated with cyclophosphamide, particularly those with breast cancer [3-6]

Ifosfamide is a chemotherapeutic drug used to treat several types of cancer, such as bladder cancer, ovarian cancer, small cell lung cancer, and osteosarcoma. Some patients have experienced visual disturbances and conjunctivitis [7].

Busulfan infusion has been associated with keratoconjunctivitis sicca in patients undergoing treatment for chronic myeloid leukemia and other myeloproliferative diseases [5, 8].

### 2. Antimetabolites

5- Fluorouracil (5-FU), a fluoropyrimidine analog, is a widely used chemotherapeutic agent for skin, gastric, intestinal, cervical, breast, and head and neck cancers. Studies have documented its association with epiphora due to canalicular stenosis [6, 9].

Capecitabine, used for colorectal cancer, gastric cancer, and breast cancer, often in combination with docetaxel, has been reported to cause adverse reactions on the ocular surface, including decreased visual acuity, corneal deposits, eye irritation, tear duct stenosis, conjunctivitis, and blepharitis [10].

Methotrexate, a chemotherapeutic and immunosuppressive agent, is utilized for various cancers and autoimmune diseases. It can be administered either orally or parenterally. It is an antagonist of folic acid. Its effectiveness has been proven in treating breast cancer, head and neck cancer, osteosarcoma, acute leukemia, and others. Ocular surface toxicity, such as decreased lacrimation reflexes [5], optic nerve and retinal changes, conjunctivitis, photophobia, cataracts, and excessive lacrimation, has been associated with methotrexate use [5, 11, 12].

Pemetrexed, administered intravenously, is used as monotherapy for advanced or metastatic non-small cell lung cancer or in combination with cisplatin for unresectable malignant pleural mesothelioma. Studies have reported that 1% to 5% of patients experienced increased lacrimation and ocular surface conditions, including conjunctivitis, as adverse reactions to pemetrexed [13].

Similarly, pentostatin, a purine analog, is used to treat chronic lymphocytic leukemia cutaneous and T-cell lymphoma. However, it is important to note that adverse reactions on the ocular surface, such as epiphora, conjunctivitis, and dry eye, have been observed in patients receiving pentostatin therapy [14].

### 3. Mitotic inhibitors

Paclitaxel and Docetaxel are members of the taxane family and are commonly used in the treatment of various cancers. Docetaxel is frequently utilized for locally advanced breast cancer with or without metastases, non-small cell lung, gastric, prostate, and head and neck cancer [15-17]. Docetaxel increased the survival rate in patients with advanced breast cancer. Several authors described the occurrence of narrowing and/or obstruction of the nasolacrimal canaliculus in breast cancer patients undergoing docetaxel treatment [18-20]. Additionally, studies have shown that epiphora (excessive tearing) is more prevalent in patients receiving weekly docetaxel treatment compared to those on a three-week treatment schedule [21, 22].

Vincristine and vinblastine act by inhibiting metaphase and impeding the development of the mitotic axis. These drugs are recommended in combination with other antineoplastic agents for the treatment of Hodgkin's disease, non-Hodgkin malignant lymphomas, Ewing's sarcoma, malignant melanoma, breast cancer, neuroblastomas, and rhabdomyosarcomas. These are administered only intravenously, and the neurotoxicity is directly proportional to the administered dose [23, 24].

### 4. Antibiotics

Doxorubicin, an anthracycline antibiotic, is commonly employed with other chemotherapeutic agents for treating various cancers, including ovarian cancer, non-Hodgkin's lymphoma, sarcoma, breast cancer, and acute leukemia. Ocular surface side effects frequently observed with doxorubicin treatment include conjunctivitis and epiphora (excessive tearing) [25].

### 5. Hormonal agents

Hormone therapy with Tamoxifen is commonly employed in the adjuvant setting or for metastatic breast cancer. There have been reported cases of keratopathy on the ocular surface since 1987 [26, 27]. Additionally, Tamoxifen has significant side effects on the optic nerve, retina (retinopathy), and lens (cataracts) [27].

### 6. Monoclonal antibodies

Rituximab is indicated in three different diseases, namely in non-Hodgkin’s lymphoma (alone or in combinations with other drugs), chronic lymphocytic leukemia (the most common form of leukemia in adults) and rheumatoid arthritis (in cases where other treatments have been ineffective or insufficient). Rituximab is an antibody directed against the CD 20 antigen on the surface of cell B [28]. In the USA, the drug is also used in treating vulgar pemphigus, some vasculitis, and in children, it is used in treating Burkitt's lymphoma but also acute B-cell leukemia [28]. Adverse effects on the ocular surface associated with Rituximab therapy include a burning sensation, excessive tearing (lacrimation), conjunctivitis, and in some instances, vision decrease or loss [28-30].

Cetuximab is a monoclonal antibody that targets the epidermal growth factor receptor (EGFR), a protein overexpressed in cancer cells. Cetuximab is indicated for colorectal cancer with metastases, non-small cell lung cancer with metastases, and head and neck cancer with metastases by inhibiting tumor growth and the development of metastases. Bilateral corneal erosions have been reported in colorectal cancer patients treated with cetuximab [30, 31].

Bevacizumab, another monoclonal antibody, acts by inhibiting vascular endothelial growth factor (VEGF). It is usually used in combination with other antineoplastic drugs in the treatment of advanced colorectal cancer or with metastases, advanced lung cancer or metastases other than the one with small cells, advanced kidney cancer and/or metastatic, in stage four glioma, relapsed or metastatic cervical cancer, and advanced liver cancer [32, 33]. While cases of ocular hyperemia at the ocular surface have been reported, adverse reactions in the posterior pole of the eye are of greater significance [33].

### 7. Drugs targeting signal transduction

Everolimus is a drug used in the prevention of transplant rejection but also in malignant tumors. Thus it has indications in breast cancer HER 2 negative, pancreatic cancer, gastrointestinal neuroendocrine tumors, and advanced kidney cancer [34].

Vemurafenib is indicated in advanced melanoma with unresectable or metastatic BRAF mutation. There is a risk of developing skin cancers with squamous cells. Intense photosensitization has been described as a secondary ophthalmological reaction to this drug. Additionally, cases of uveitis and retinal vein occlusion have been reported. Patients should be monitored during treatment with this medicine in order to quickly notice the appearance of such an ophthalmological side effect and to be able to intervene timely. [35-38].

## CONCLUSION

Chemotherapeutic drugs act mainly on cancer cells that divide rapidly but can also affect normal cells in the body, leading to side effects that should not be overlooked. Even if significant progress has been made in developing antineoplastic medication, mortality due to cancer is still increased, the second after cardiovascular diseases.

The delicate homeostasis of the ocular surface is affected by the cytotoxicity of these drugs, as evidenced by numerous reported cases in the specialized literature. Factors such as dosage, rate of administration, cumulative dose, and method of drug delivery have been shown to influence the occurrence, frequency, and intensity of ocular adverse effects.

The ophthalmologist must also be a part of the oncology patient's team. Oncology patients must undergo ophthalmology consultations before initiating antineoplastic treatment and regularly during treatment. This allows the ophthalmologist to promptly manage any adverse ophthalmological reactions that would decrease the patient's quality of life. It is important to note that most ocular side effects are temporary and resolve after discontinuing antineoplastic treatment.

Reporting every adverse ophthalmological reaction during chemotherapeutics is essential to accurately analyze these drugs.
